# Performance of Straw/Linear Low Density Polyethylene Composite Prepared with Film-Roll Hot Pressing

**DOI:** 10.3390/polym12040860

**Published:** 2020-04-09

**Authors:** Lei Zhang, Huicheng Xu, Weihong Wang

**Affiliations:** Key Lab of Bio-based Material Science and Technology (Ministry of Education), College of Material Science and Engineering, Northeast Forestry University, Harbin 150040, China; zl857910712@163.com (L.Z.); Hynner@163.com (H.X.)

**Keywords:** bio-composite, linear low density polyethylene, performance, straws

## Abstract

Thermoplastic composites are usually prepared with the extrusion method, and straw reinforcement material must be processed to fiber or powder. In this study, film-roll hot pressing was developed to reinforce linear low density polyethylene (LLDPE) with long continuous straw stems. The long straw stems were wrapped with LLDPE film and then hot pressed and cooled to prepare straw/LLDPE composite. Extruded straw fiber/LLDPE composite was prepared as a control. The mechanical properties of these LLDPE-based composites were evaluated. The hot pressed straw/LLDPE composite provided higher tensile strength, tensile modulus, flexural strength, flexural modulus, and impact strength than the traditional extruded straw/LLDPE composite, by 335%, 107%, 68%, 57%, and 181%, respectively, reaching 35.1 MPa, 2.65 GPa, 3.8 MPa, 2.15 GPa, and 25.1 KJ/m^2^. The density of the hot pressed straw/LLDPE composite (0.83 g/cm^3^) was lower than that of the extruded straw/LLDPE composite (1.31 g/cm^3^), and the former had a higher ratio of strength-to-weight. Scanning electron microscopy indicated that the orientation of the straws in the composite was better with the new method. Differential scanning calorimetry tests revealed that in hot pressed straw/LLDPE composite, straw fibers have a greater resistance to the melting of LLDPE than extruded composite. Rotary rheometer tests showed that the storage modulus of the hot pressed straw/LLDPE was less affected by frequency than that of the extruded composite, and the better elastic characteristics were pronounced at 150 °C. The hot pressed straw/LLDPE composite absorbed more water than the extruded composite and showed a potential ability to regulate the surrounding relative humidity. Our results showed that straw from renewable sources can be used to produce composites with good performance.

## 1. Introduction

Natural fiber reinforced composite is a kind of biomass composite. It uses plant-based natural fibers (such as straw, bamboo, jute, and sisal, etc.) as reinforcement, polymers such as polyethylene (PE) and polypropylene (PP) as the matrix, prepared by blending extrusion or molding [1,2]. Natural fiber reinforced composites are widely used in automotive interiors, panel or wall panels, and sports equipment because of their low cost, safety, non-toxicity, renewability, wide source and excellent performance [3,4].

Straw is a type of renewable, abundant natural fiber. The world’s crops can provide approximately two billion tons of straw per year [5]. In the past, the main deposal method of straw was incineration, but given the serious associated environmental problems and increasing environmental awareness, straw recycling has become an international priority [6]. 

Researchers have made some achievements in straw fiber reinforced composites. Nyambo et al. [7] used maleic acid-grafted polyurethane (PU-g-MA) to improve the interfacial adhesion between wheat straw and polyurethane (PU). They found that the addition of 3 phr and 5 phr PU-g-MA significantly increased the tensile strength (20%) and flexural strength (14%) of straw/PU composites, and proved that the increase in strength was due to the well combination of fibers and matrix. Xiao et al. [8] treated the straw with NaOH solution, blended the straw, polyethylene, stearic acid and maleic anhydride, then hot pressed to manufacture the straw/PP composite. The composite has low water absorption and good acid and alkali resistance; Zhang et al. [9] investigated the effects of different straw treatment methods, the particle size of straw powder, and the mass fraction of straw on the mechanical properties of straw/PP composites. The results show that when the straw is treated with the silane coupling agent KH570, the mechanical properties of the straw/PP composite are the best when the particle size of the straw powder is 60 mesh and the mass fraction is 50%; Zabihzadeh et al. [10] investigated the effect of maleic acid grafted polyethylene on the mechanical properties of straw/high density polyethylene (HDPE) composites. It was found that compared with no addition, adding 2% MAPE can increase the tensile strength of the composite by 43%, increase the tensile modulus by 116%, and increase the impact strength by 12%. Even with the addition of 1%, there is a clear improvement.

Straw fiber reinforced composites are a branch of Wood Plastic Composites (WPC). The extrusion molding process, one of the main molding processes of WPC, refers to a processing method in which natural fiber powder, thermoplastic, and various additives are melted in a high temperature, high pressure extruder to be fully mixed, plasticized, finally passed through the top mold of the machine continuously. Due to its advantages of continuous production and high production efficiency, extrusion molding is widely used in industrial production. As of 2017, China’s WPC output was close to three million tons, accounting for two-thirds of the world’s total output, and the China’s production, consumption and exports ranked first in the world, which is precisely due to the development and improvement of extrusion molding processes over the years. The extrusion molding process is mainly divided into one-step extrusion and two-step extrusion. In two-step molding process, the raw materials are first pelletized in a twin-screw extruder, then extruded in single-screw extruder to prepare the composite, and the one-step process skips the pelletizing stage. The two-step process is simpler, flexible, and easy to adjust, which is the most commonly used molding process for enterprises and research units. However, the extrusion molding is easily affected by many factors due to the complicated process. The combined effect of many factors caused many uncertainties among the variables [11].

In extrusion molding process, straw is mostly used in form of short fiber or powder in composite structures. The interface bonding conditions of long fiber reinforced composites are very different from that of short fiber reinforced composites [12]. For example, the friction at the fiber/plastic interface of the former is much larger than that of the latter [13]. The stem of the straw itself has good tensile strength. Nevertheless, this strength often decreases when the straw is used in powder form. We know that orienting short fibers and powders is difficult. Therefore, owing to long straw integrity, long straw reinforced composites should theoretically have high strength. In addition, polypropylene (PP) and high density polyethylene are commonly used as matrixes because of their high strength and suitable processing temperatures [14,15], but the toughness of HDPE and PP-based composites is poor [16]. Therefore, other matrix options must be developed. Linear low density polyethylene (LLDPE) has excellent properties, such as tear strength and environmental stress crack resistance, in addition to the properties of general polyolefins [17]. LLDPE has a disadvantage of poor stiffness besides, it potentially could be remedied by straws.

The main aim of this study is to develop a novel method to prepare long straw stem reinforced LLDPE composites, whose properties are compared with those of short fiber reinforced LLDPE composites.

## 2. Materials and Methods

### 2.1. Materials

Straws were obtained from the suburb of Harbin, China. LLDPE film (Film, 10H01) and LLDPE particles (Hytrel, 22402) were purchased from Runwen Packaging Materials Co., Ltd., Shanghai, China. Talcum powder (2000 mesh) was produced by Liangjiang Titanium Chemical Products Co., LTD, Shanghai, China, and was used to enhance the stiffness of straw-plastic composite. Maleic anhydride grafted polyethylene (MAPE) with a grafting rate of 0.9% was purchased from Rizhisheng Fine Chemical Co., Ltd., Nantong, China, and was used as the coupling agent. PE wax, also from Shanghai Liangjiang Titanium Chemical Co., Ltd. and zinc stearate, purchased from Natural Oil Chemical Co., Ltd., Pasir Gudang Town, Johor Bahru, Malaysia, were used as lubricants.

### 2.2. Preparation of Straw/LLDPE Composite

The straw was first oven dried at 103 °C in a DHG-9140A drier (Yiheng Scientific Instrument Co., Ltd., Shanghai, China) to decrease its moisture content to below 3%. Then the straw was used to prepare composite through two molding processes. 

#### 2.2.1. Extrusion Molding 

Dry straws were cut to 1 cm lengths. Then the short straws, LLDPE particles, talcum, MAPE, PE wax and zinc stearate were weighed in a ratio of 60:25:10:3:1:1. These materials were mixed in a SHR-10A high-speed mixer (Tonghe Rubber & Plastic Machinery Co., Ltd., Zhangjiagang, China) for 5 min. The mixture was pelleted in a JSH30 twin-screw extruder (Nanjing Rubber & Plastic Machinery Factory in Nanjing, China) at 140 °C and then shattered in a GL-01 pulverizer (Evian Machinery Co., Ltd., Shanghai, China). The pulverized raw materials were fed into a BHMS single-screw extruder (Nanjing Saiwang Technology Development Co., Ltd., Nanjing, China). The extruded lumber had a rectangular cross section of 40 mm in width and 4 mm in thickness.

#### 2.2.2. Film-Roll Hot Press Molding

**Figure 1:**
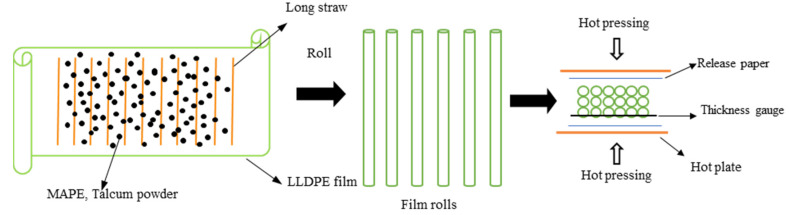
Preparation of straw/LLDPE composite through the film-roll hot pressing process.

**Figure 2:**
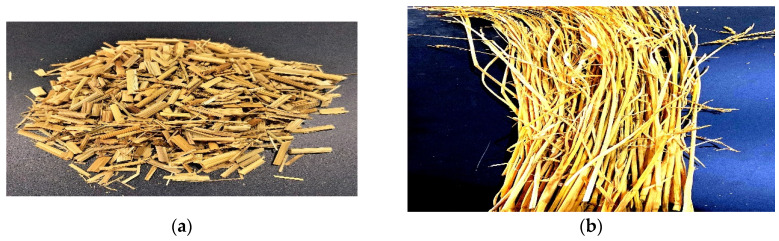
Straw particle (**a**), straw stem (**b**).

MAPE and talcum powder were evenly spread on the LLDPE film. Long dry straws were spread in parallel on the film. The proportions of straw, LLDPE, talcum and MAPE were 60%, 27%, 10% and 3%, respectively. The film was rolled up, and the straw stems were enveloped. The rolls were first pre-heated without pressure for 4 min and then hot pressed for 5 min under 10 MPa pressure in a SY01 hot press (Shanghai Board Equipment Technology Co., Ltd., Shanghai, China). In this process, the temperature was set to 140 °C. After hot pressing, the panel was cooled. A gauge was used to control the thickness of the straw/LLDPE composite. The size of the result panel was 165mm × 165mm × 4 mm. The detailed steps are shown in Figure 1. Straw particles and straw stems are shown in Figure 2.

### 2.3. Characterization of Straw/LLDPE Composite

#### 2.3.1. Mechanical Property Tests

The unnotched impact strength was examined on the basis of GB/T 1043.1–2008 (“Plastics, Determination of Charpy Impact Properties, Part 1: Noninstrumented Impact Test”) with a JC-5 Charpy Impact Tester (Chengde Precision Testing Machine Co., Ltd., Chengde, Hebei, China). Specimens of 80mm × 10mm × 4 mm with a span length of 60 mm were analyzed. The striking velocity of the tests was 2.9 m/s, and the pendulum energy was 2 J. Six replicates of each preparation were tested to determine the impact strength.

The tensile tests were carried out in accordance with the method of GB/T 1040.2–2006 (“Plastics–Determination of tensile properties–Part 2: Test conditions for molding and extrusion plastics”). The test piece had a dumbbell shape, and its gauge length was 50 mm. The length of the specimen is parallel to the fiber orientation. The length of the test piece was 165 mm, the width of the narrow portion was 13 mm, and the thickness was 4 mm. The tests were carried out with a loading speed of 5 mm/min. The clamp stretches the specimen along the fiber orientation. Six replicates of each preparation were tested to obtain values for the tensile modulus and strength.

Flexural tests were carried out in accordance with the procedure of GB/T 1449–2005 (“Fiber-Reinforced Plastic composite- Determination of Flexural Properties”). The specimens of 80mm × 13mm × 4 mm had a span length of 64 mm. A loading speed of 2 mm/min was used for testing. The extension direction of the probes is perpendicular to the fiber orientation. Six replicates of each preparation were tested to obtain values for the flexural modulus and flexural strength. The tensile and flexural tests are completed by a CMT5504 mechanical testing machine (MTS industrial Systems (China) Co., LTD., Shanghai, China).

#### 2.3.2. Density Tests 

The composites were cut into 50 mm × 35 mm test pieces, and the length, width and thickness of the sample and the sample quality were measured according to GB/T 17657–2013 “Physical Testing Methods for Artificial Board and Finished Panels.” The composite density was characterized according to the ratio of mass to volume. Three replicates of each preparation were tested. The density of each replicate was measured once, then averaged the measurement of each replicate.

#### 2.3.3. Water Absorption Performance

The material was formed into a test piece of 76.2mm × 25.4mm × 4 mm, and water absorption performance tests were carried out in accordance with the standard ASTM D570 “Standard Test Method for Water Absorption of Plastics.” The test pieces were dried in an oven for 24 h at 50 °C and then completely immersed in water at 24 °C. After 24 h, the test pieces were removed and weighed immediately. Vernier calipers were used to measure the thickness of test pieces before and after soaking. Three replicates of each preparation were tested. The mass and thickness of each replicate were measured three times and then averaged.

The water absorption and thickness expansion were calculated according to Equations (1) and (2), respectively:

(1) $$c=\frac{m_{1}-m_{0}}{m_{0}^{}}\times 100\%$$

(2) $$T=\frac{t_{1}-t_{0}}{t_{0}^{}}\times 100\%$$

where *c* denotes the water absorption mass fraction, *T* denotes the thickness expansion ratio, *m*_0_ and *t*_0_ denote the mass and thickness of the test piece after drying, respectively, and *m*_1_ and *t*_1_ denote the mass and thickness of the test piece after immersion, respectively.

#### 2.3.4. Differential Scanning Calorimetry (DSC) Analysis 

A differential scanning calorimeter (DSC Q100, TA Instruments, New Castle, PA, USA) was used to detect the melting behavior of the straw/LLDPE composite. The temperature of the straw/LLDPE composite sample was reduced to −70 °C, then heated to 180 °C at a rate of 10 °C min^−1^. The entire testing process was performed under a nitrogen atmosphere. 

#### 2.3.5. Interfacial Morphological Observations by Scanning Electric Microscopy (SEM)

SEM was used to characterize the internal structural changes. The straw/LLDPE composite samples were frozen in liquid nitrogen for 10 min and then broken. The broken surfaces were sputter-coated with gold and then observed under a scanning electron microscope (FEI Quanta 200, FEI Co, Hillsboro, TX, USA) operated at an acceleration voltage of 12.5 kV. Three samples of each preparation were observed.

#### 2.3.6. Rotating Rheological Tests

The dynamic rheological properties of straw/LLDPE composite were tested with a rotary rheometer (AR2000ex, TA Instruments, New Castle, PA, USA). Dynamic frequency scanning tests were conducted. The frequency range was 628.3 rad/s to 0.01 rad/s, and the strain was fixed at 0.05%. The above operations were carried out at 150 °C.

## 3. Results

### 3.1. Mechanical Property Analysis

**Figure 3:**
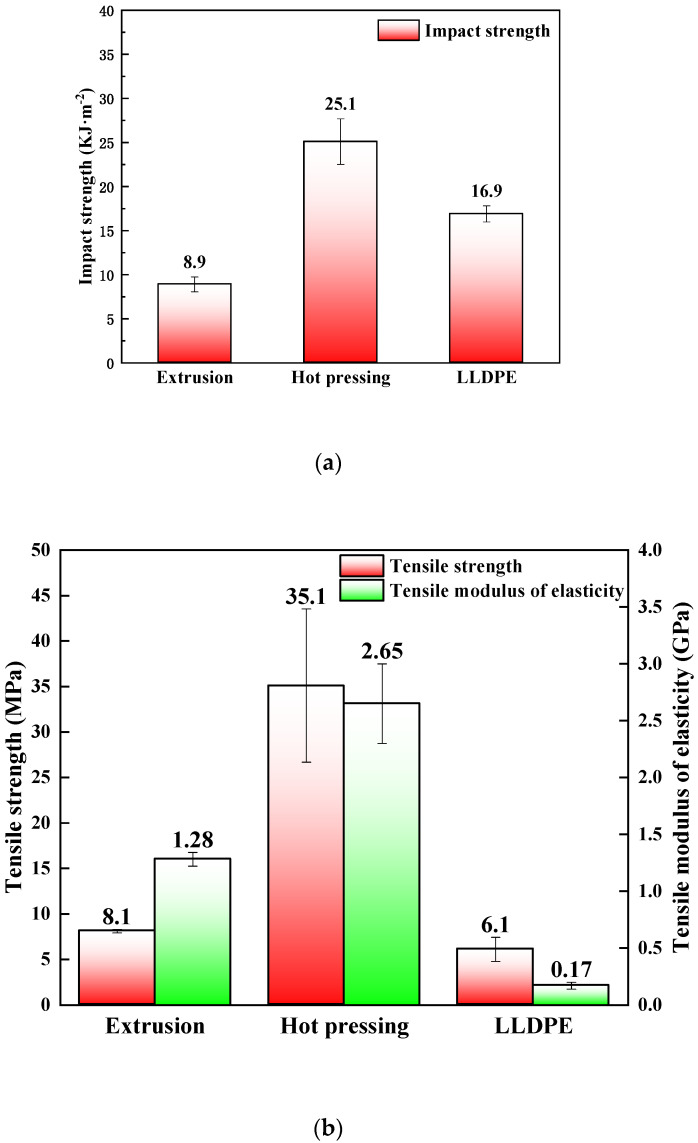

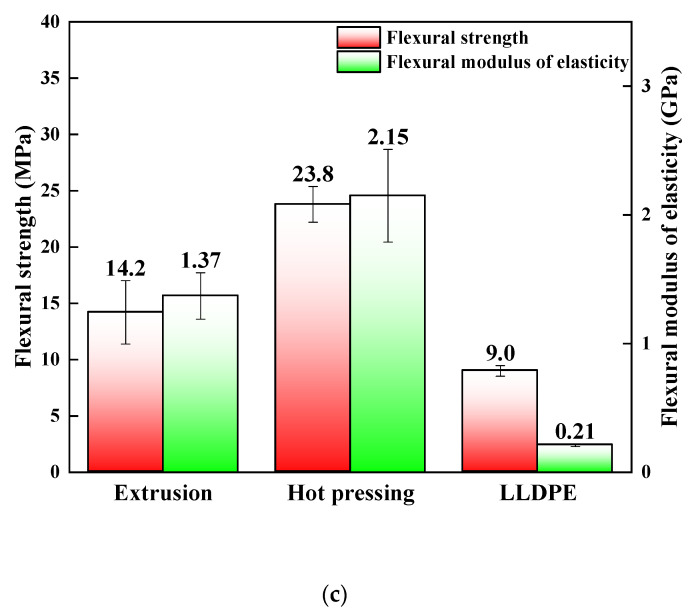
Impact strength (**a**), tensile properties (**b**) and bending properties (**c**) of hot pressed and extruded straw/LLDPE composite.

The mechanical properties of extruded and hot pressed straw/LLDPE composite are shown in Figure 3.

As shown in Figure 3, the mechanical properties of hot pressed straw/LLDPE composite were significantly higher than those of the extruded composite. Compared with the extruded panel, the hot pressed panel showed 181% greater impact strength (Figure 3a), 335% greater tensile strength, 107% greater tensile modulus (Figure 3b), 68% greater bending strength and 57% greater flexural modulus (Figure 3c). These results might be due to several factors.

Fiber orientation is known to positively influence the strength of reinforced composites. In the extruded straw/LLDPE composite, the straw fibers were randomly distributed, whereas in the hot pressed straw/LLDPE composite, the straw stems were highly oriented and remained at full length. Under loading, more energy was needed to overcome the interface bonding between the straws and the LLDPE as well as to break the straw stems themselves [18]. However, in extrusion preparation, the straw was in the forms of short fibers or powder, whose specific ratios were small and whose lengths were shorter than the critical length. If the length is less than the critical length, straw fibers are not be snapped but may be pulled out (the fibers slip from the matrix), i.e., straw fibers fail to fully exert the fiber strength within critical length and cannot play a reinforcing role (only as a filling material).

Because the length of straw stems is longer than straw fibers, the energy consumed for fiber extraction in the composite is higher, so the impact strength of hot pressed composite is higher. In addition, the fiber ends where stress is concentrated is the crack initiation point. The longer the fiber, the fewer the fiber ends in the composite. This is also the reason for its high impact strength.

### 3.2. Melting Performance Analysis

**Figure 4:**
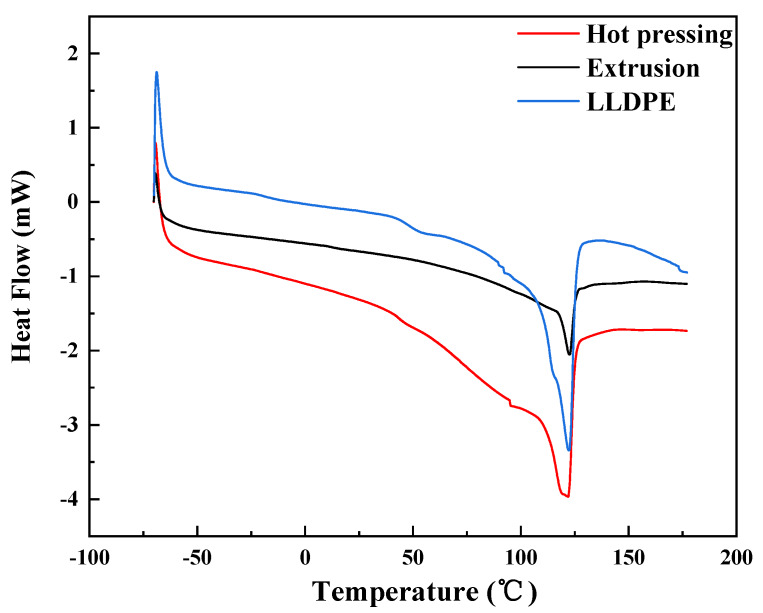
DSC image of hot pressed and extruded straw/LLDPE composite.

The melting performance of extruded and hot pressed straw/LLDPE composites is shown in Figure 4. The crystallinity of LLDPE was calculated according to Equation (3):

(3) $$X_{C}=\frac{\Delta H_{f}(m_{c}/m_{LLDPE})}{\Delta H_{f}^{0}}$$

where $\Delta H_{f}$ denotes the melting enthalpy, $m_{c}$ denotes the mass of the sample, $m_{LLDPE}$ denotes the mass of LLDPE in the sample and $\Delta H_{f}^{0}$ denotes the melting enthalpy of LLDPE with 100% crystallization, 293 J/g [19].

The data in Table 1 show the enthalpy and melting temperatures of samples. The composite manufactured by hot pressing and extrusion showed clear melting peaks at 121.97 °C and 122.61 °C, respectively, values similar to that of LLDPE, at 122.28 °C. The endothermic peaks might possibly be caused by the melting of LLDPE [20]. The peak of the hot pressed composite was wider and higher than that of the extruded composite. As shown in Table 1, the melting enthalpy of the hot pressed straw/LLDPE composite was much higher than that of the extruded composite. This shows that in hot pressed straw/LLDPE composite, straw fibers have a greater resistance to the melting of LLDPE, so that the melting process requires more heat than extruded composite [21]. This is because the melting enthalpy is related to the crystallinity. Compared with long straw stems, short straw fibers are more easily dispersed uniformly in the composite, and the contact area with LLDPE molecules increases, which plays a role in diluting LLDPE, so it can reduce the interaction between LLDPE molecules to a greater extent. The lower crystallinity, the fewer the heat required for heating and melting, i.e., the lower melting enthalpy. Pore structure of long straw in hot pressed composite would be expected to store more thermal energy, thereby resulting in slow progress of melting. 

### 3.3. Water Absorption Performance Analysis

**Figure 5:**
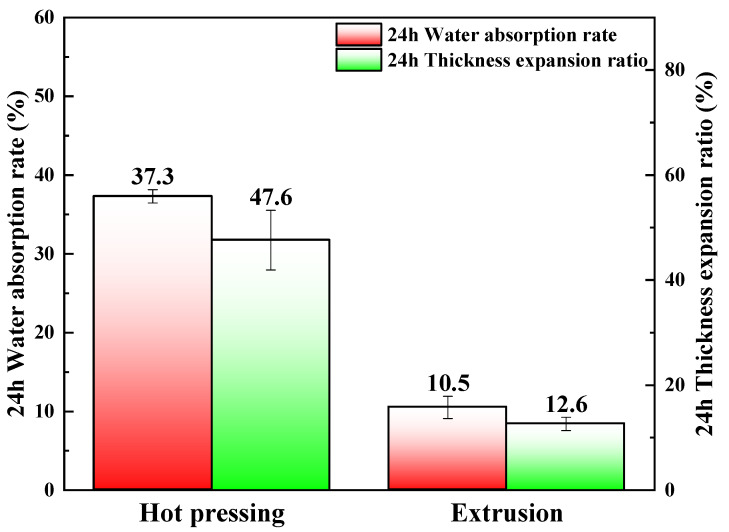
Water absorption mass fractions and thickness expansion ratios of hot pressed and extruded straw/LLDPE composite (immersed for 24 h).

The water resistance test results of hot pressed and extruded straw/LLDPE composites are shown in Figure 5.

Both the water absorption rate and the thickness expansion ratio of hot pressed straw/LLDPE composite were higher than those of the extruded composite, because in the hot pressing process, LLDPE matrix cannot completely fill in the cavities of the straw stem, and consequently the area of the straw contacting water is larger. In the extrusion process, almost all surfaces of the tiny straw particles were covered with LLDPE matrix [22]. Evenly distributing the raw material can effectively improve water resistance [23]. Possible effective measures include breaking the stem along the length of straw, which can take advantage of long fibers and also facilitate LLDPE matrix to enter the straw cavity during hot pressing, reducing the contact area between straw and water. In addition, a layer of LLDPE can be coated on the outside before hot pressing to seal the material.

### 3.4. Morphology of the Fracture Surface

#### 3.4.1. Interface Bonding 

**Figure 6:**
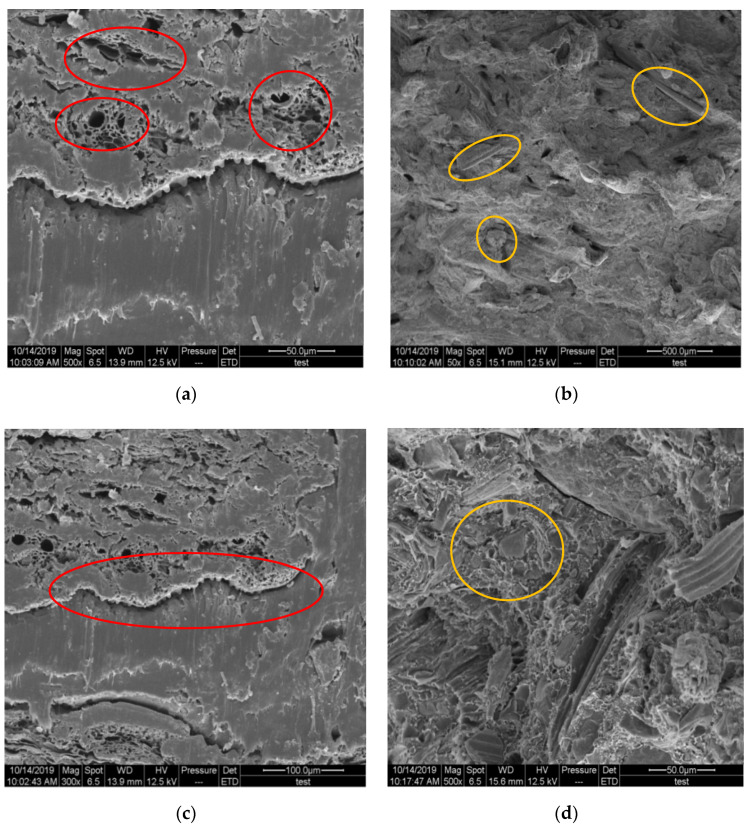
Microscopic morphology of the fracture surface of hot pressed (**a**,**c**) and extruded (**b**,**d**) straw/LLDPE composite.

The interfacial bonding and fiber orientation of the straw/LLDPE composite manufactured through the two methods were characterized by SEM (Figure 6).

In the hot pressed composite, only the cross section of the straw could be seen (circles in Figure 6a), thus indicating that the direction of the straw was well fixed. In Figure 6b, the circled straw fibers in the extruded composite are randomly distributed. As further shown in circles of Figure 6c, a crack is present at the interface location between the straw and the LLDPE matrix in the hot pressed composite. In contrast, the straw and LLDPE matrix are relatively tightly combined in the extruded composite (circles in Figure 6d). In addition, the hollow structure of the straw in the hot pressed composite is not filled, thereby contributing to the higher impact strength. The holes left by straws that had been pulled out indicated that the straw fibers did not break in tensile loading (Figure 6b,d). However, the straw stems fully broke, and no holes were left in the matrix (Figure 6a,c), thus indicating that the long straw stems contributed to the strength. These structural results explained the differences in both the strength and water absorption of the composite. The hollow structures of the long straws that remained also explained the low density of the hot pressed straw/LLDPE composite (Table 2) [24]. In addition, the extrusion process can uniformly mix the raw materials and tightly combine them, thereby increasing the density of the composite.

#### 3.4.2. Analysis of Tensile Fracture Mechanism 

**Figure 7:**
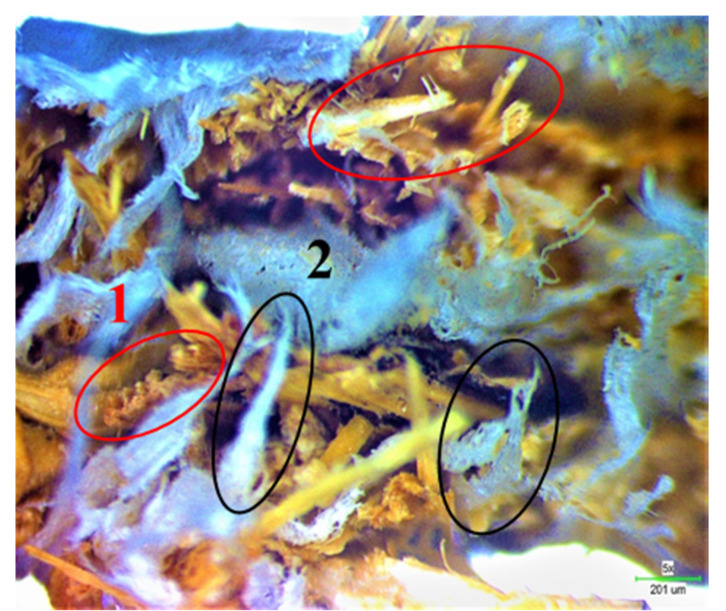
Optical micrograph of fracture surface of the hot pressed straw/LLDPE composite.

**Figure 8:**
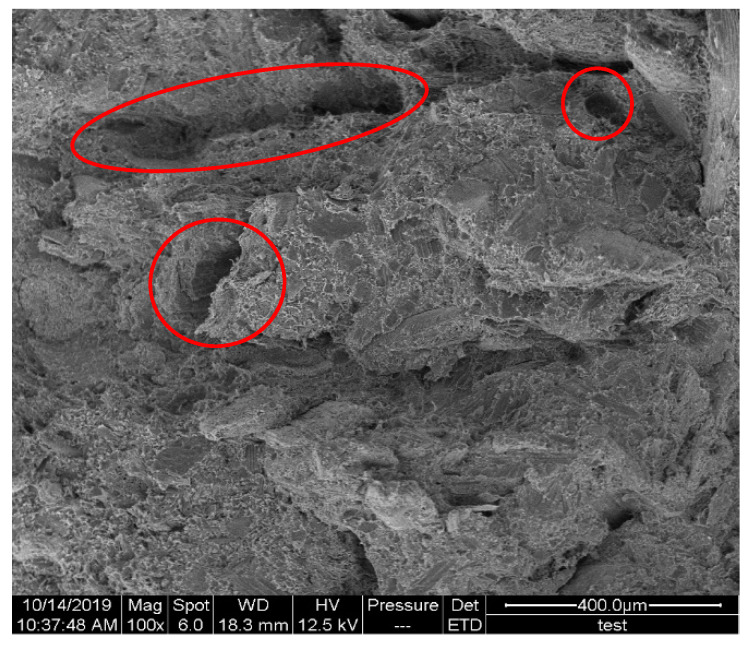
SEM of fracture surface of the extruded straw/LLDPE composite.

In the straw/LLDPE composite, the main fracture forms are fiber fracture, interface detachment and matrix fracture. In Figure 7, the broken straw fiber (Circle 1) and the tip-shaped LLDPE (Circle 2) can be seen. In Figure 8, we can see the holes (The circled part) left by short fibers pull-out, indicating that the strength of the straw itself is not fully exerted in the extruded composite.

**Figure 9:**
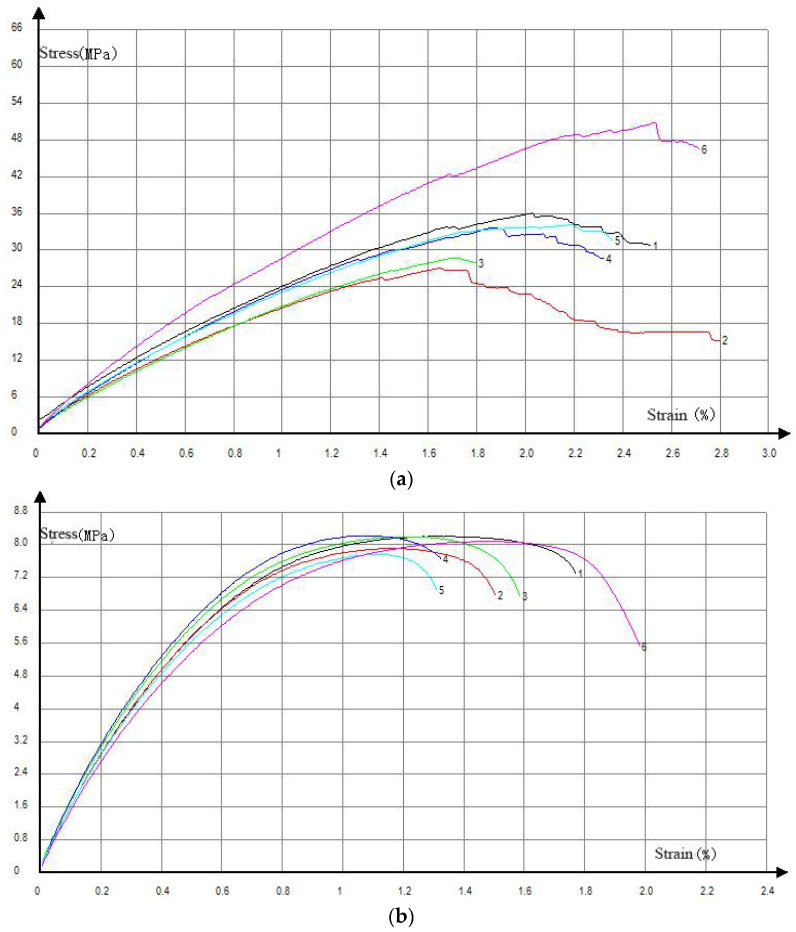
Tensile stress-strain curves of specimen 1 to 6 of hot pressed (**a**) and extruded (**b**) straw/LLDPE composite.

Tensile stress-strain curve of hot pressed and extruded straw/LLDPE composites is shown in Figure 9. Comparing Figure 9a,b, it can be seen that the stress-strain curve of the extruded composite is relatively smooth, thus the fracture process is relatively gentle, which indicates that the tougher LLDPE plays a major bearing role. Therefore, combining Figure 8 and Figure 9b shows that the mechanical strength of the extruded composite mainly comes from the LLDPE matrix. In the curve image of the hot pressed composite, the stress drops suddenly. This is due to the sudden separation of the straw fiber and LLDPE matrix interface and the sudden breaking of the straw. Combining Figure 7 and Figure 9a, it can be known that the mechanical strength of the hot pressed composite mainly comes from the combination of the fiber matrix and the strength of the straw, and the matrix itself has less effect.

### 3.5. Dynamic Rheological Performance Analysis

**Figure 10:**
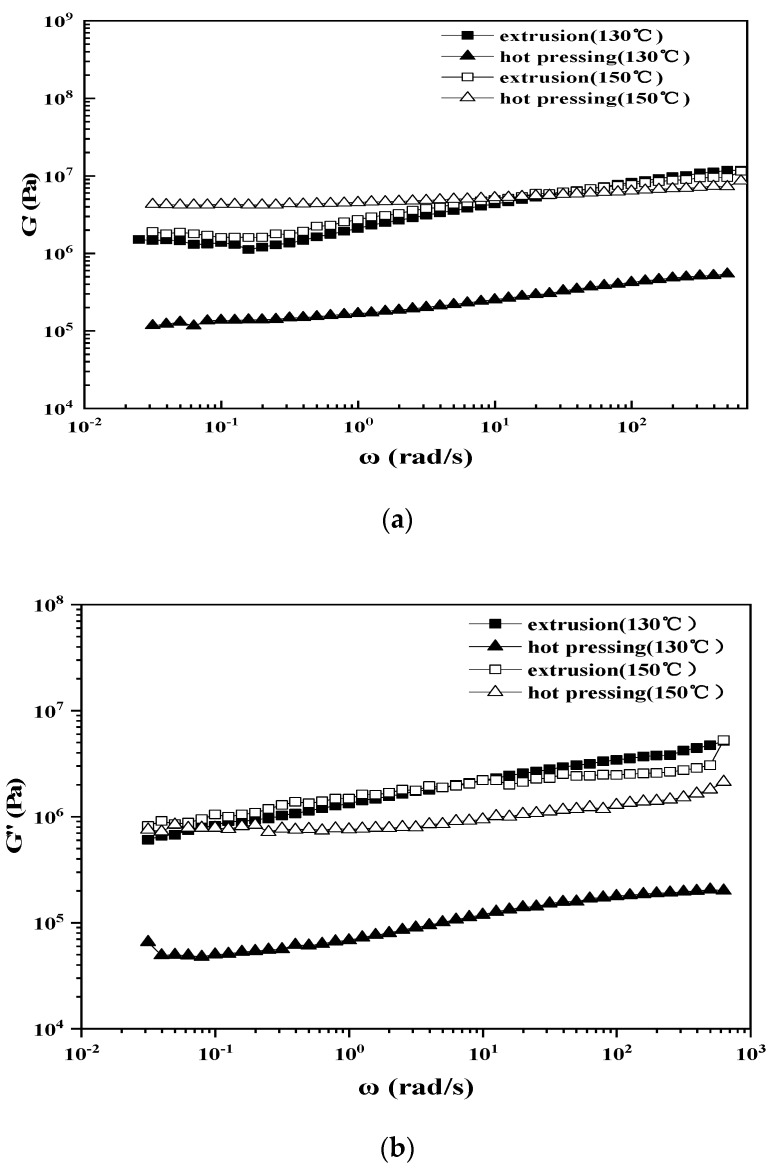

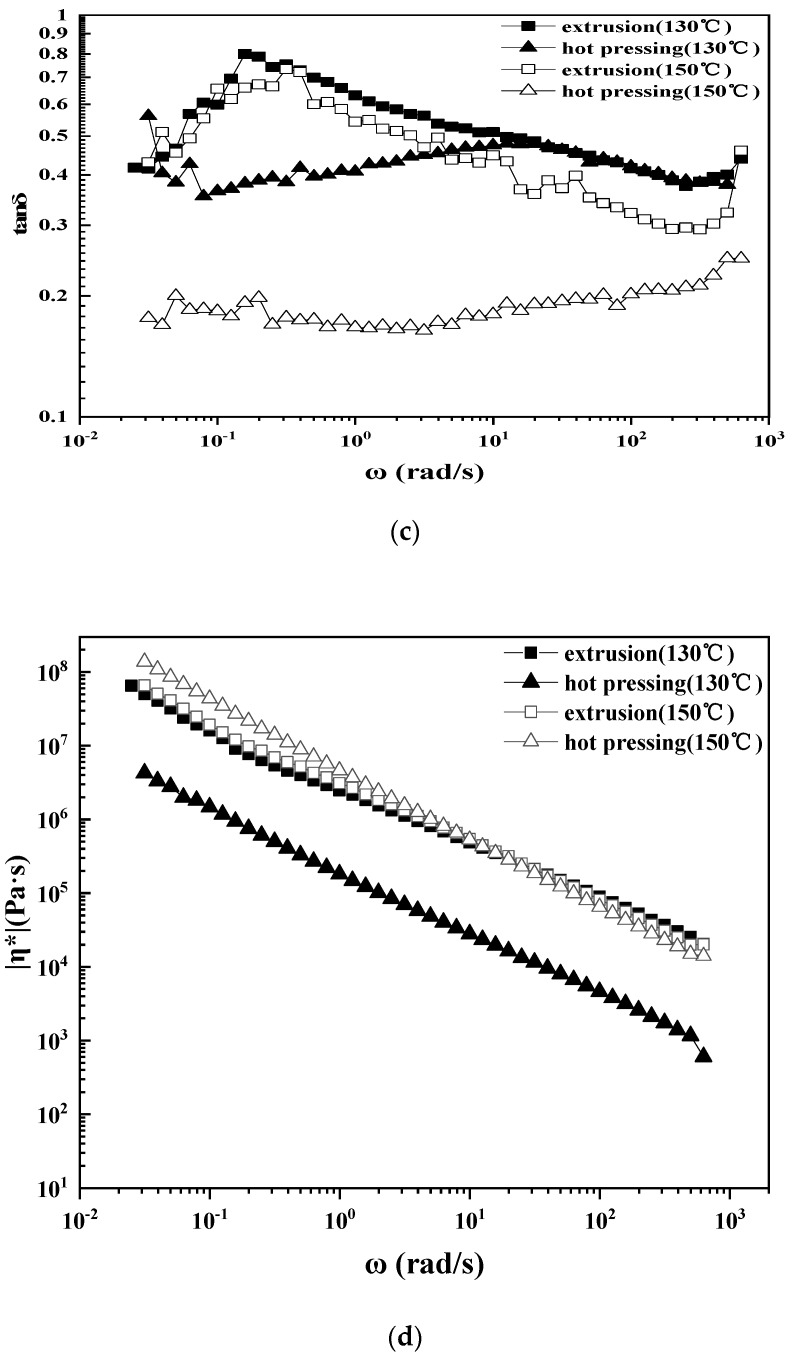
Plots of the storage modulus (**a**), loss modulus (**b**), loss tangent value (**c**) and complex viscosity (**d**) vs ω of straw/LLDPE composite.

Plots of the storage modulus, loss modulus, loss tangent value and complex viscosity vs ω of straw/LLDPE composite is shown in Figure 10. Figure 10a shows the relationship between the storage modulus (G′) and the angular frequency. By increasing the test temperature (from 130 °C to 150 °C), a large increase in storage modulus can be seen for the hot pressed composite, which is not the case for extruded composite. This may be because at a temperature closer to the *T*_m_ of LLDPE (*T*_m_ of LLDPE measured by DSC is 122 °C), the LLDPE melt is still relatively hard and the molecular chain flexibility is poor. In this case, the uneven fiber distribution has a negative effect on the deformation of the matrix. In contrast to the changes in G′ of hot pressed straw/LLDPE composite with frequency, a modulus platform in the low frequency region was observed for the extruded straw/LLDPE composite. This phenomenon, so-called solid-like behavior [25,26,27], occurs because of the formation of three-dimensional ordered structures such as agglomerates, skeletons and networks inside the system [28]. The viscoelastic behavior of the low ω region is the motion response of the long-chain segment of the polymer or even the entire macromolecular chain, and the three-dimensional ordered structure limits the long-term movement of macromolecular motion units [25,26,29]. The hot pressed straw/LLDPE composite showed very little change as the frequency increased. The slight modulus increase occurred because the parallel straws acted as a skeleton and prevented the LLDPE from sliding. Similarly, as shown in Figure 10b, both the hot pressed and extruded straw/LLDPE composites showed stable loss modulus with frequency variation.

Figure 10c shows the relationship between the loss tangent value of the straw/LLDPE melting and the angular frequency. The loss tangent value is the ratio of the loss modulus to the storage modulus. As shown in Figure 10c, the tan δ of the extruded straw/LLDPE melt showed a sharp peak at around 0.2 rad/s, whereas the hot pressed straw/LLDPE melt did not show a clear peak. In the G′—ω curve, the tanδ peak appears along with the modulus platform; that is, the tanδ peak is also a characteristic of solid-like behavior [30]. The change in the value of tanδ indicates a change in viscoelasticity, thus demonstrating that the viscoelasticity of the hot pressed composite is fairly stable [31].As shown in Figure 10c, tanδ was less than 1 over the entire scanning frequency range, thus indicating that the straw/LLDPE melting exhibited elasticity. We conclude that hot pressed straw/LLDPE composite had the clearest elastic characteristics, according to its low tanδ value and high G′ value [32].

Figure 10d shows the relationship between the complex viscosity ($\left|\eta^{*}\right|$) and the angular frequency.

As shown in the figure, as the frequency increases, the viscosity shows a downward trend, i.e., the phenomenon of shear thinning occurs. This is because the viscosity is the ratio of stress to strain rate, and according to Power-Law Equation

(4) $$\sigma =K\cdot \gamma^{n}$$

We can get Equation

(5) $$\eta =\frac{\sigma }{\gamma }=K\cdot \gamma^{n-1}$$

where *η* denotes the viscosity, *σ* denotes the stress, *γ* denotes the strain rate, *K* and *n* are both constants. High-molecular polymers such as PE are pseudoplastic liquids, and the *n* value of the pseudoplastic fluid is less than 1, so when the strain rate increases, the viscosity of the system decreases. The opposite of pseudoplastic fluid is dilatant liquid, such as corn paste, whose n value is more than 1, and the viscosity will increase as the strain rate increases [33]. 

There is always a certain speed gradient between the various liquid layers when the polymer flows. If a large molecule passes through several liquid layers with different flow rates at the same time, each part of the same macromolecule must advance at different speeds. This situation obviously cannot last. Therefore, during the flow, each long-chain molecule always tries to make itself all enter the liquid layer with the same flow rate. The parallel distribution of liquid layers with different flow rates results in the orientation of the macromolecules in the flow direction, which causes the viscosity to decrease with increasing frequency during the flow. With the increase of experimental temperature, the kinetic energy of the molecule of hot pressed composite is increased, but also increases the degree of intermolecular collision, which makes the viscosity increase.

**Figure 11:**
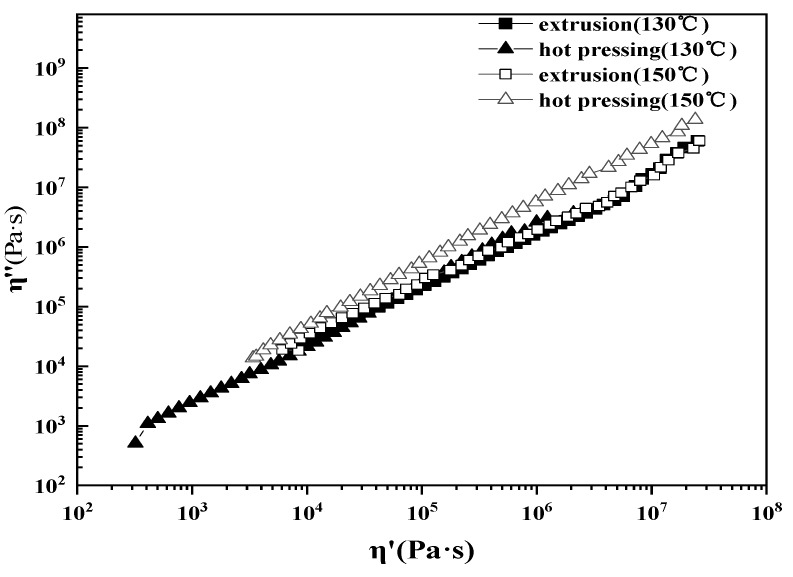
Loss viscosity of straw/LLDPE composite as a function of storage viscosity.

In dynamic tests, the relationship between dynamic viscosity and loss viscosity, the Cole-Cole curve, can give information about the various relaxation processes in heterogeneous polymer systems. As shown in Figure 11, the right end of the curve of the extruded composite is slightly upturned, so-called “tailing” phenomenon occurs. This shows that there are two relaxation mechanisms in the system of the extruded composite. This may be because LLDPE is more prone to entanglement in the extruded composite system, and this structure relaxes very slowly.

### 3.6. Physical Drawing of Extruded and Hot Pressed Straw/LLDPE Composites

**Figure 12:**
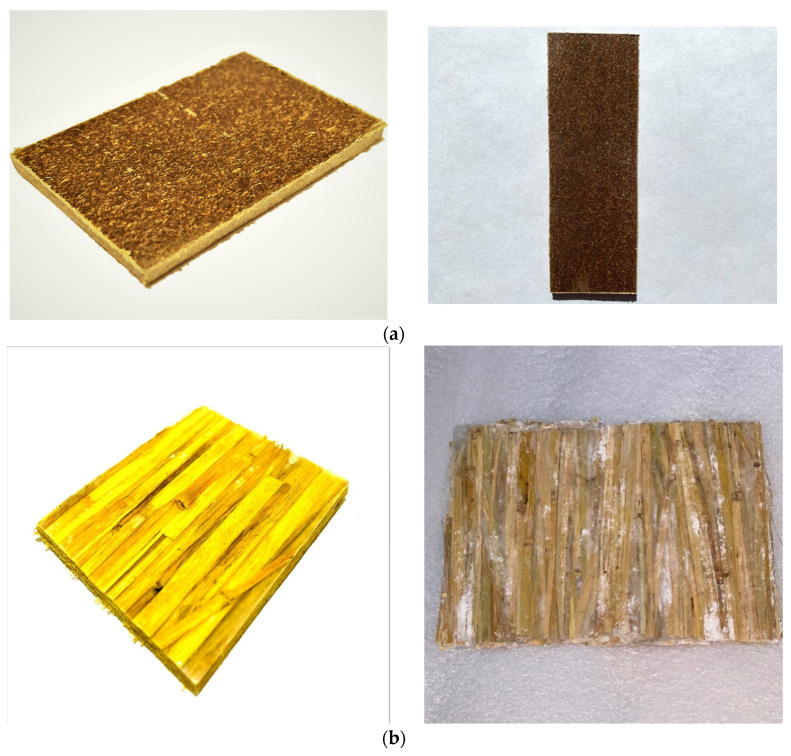
Extruded (**a**) and hot pressed (**b**) straw/LLDPE composites.

The physical picture of the extruded and hot pressed composites is shown in Figure 12.

## 4. Conclusions

This study developed a new method for constructing straw-plastic composite and compared it with conventional extrusion methods. Tests of the mechanical properties verified that the hot pressed long straw stem reinforced LLDPE composite had relatively higher strength and modulus. Microstructural observations showed better fiber orientation of the hot pressed straw/LLDPE composite, and this factor had the greatest influence on the mechanical properties of the straw/LLDPE composite. According to the results of DSC, straw fibers have a greater resistance to the melting of LLDPE in hot pressed straw/LLDPE composite, so that the melting process requires more heat than extruded composite. The results from dynamic rheological analysis indicated that the storage modulus of the straw/LLDPE melt manufactured by hot pressing was more stable than that of the extruded composite. The elastic characteristics of the hot pressed straw/LLDPE melt were more pronounced than those of the extruded composite. The hot pressed straw/LLDPE composite had higher water absorption, thus indicating its ability to regulate the surrounding relative humidity. Straw/LLDPE composites are expected to be applicable in interior decoration materials, owing to their high strength-to-weight ratio and the absence of chemical emission such as those from adhesives.

## Figures and Tables

**Table 1:** Melting enthalpy and temperature of straw/LLDPE composite.

Sample	*X*_c_ (%)	*T*_m_ (°C)	Δ*H*_f_ (J/g)
LLDPE	17.41	122.28	51.01
hot pressing	35.76	121.97	62.87
extrusion	6.94	122.61	12.20

*X*_c_—crystallinity; *T*_m_—melting temperature; Δ*H*_f_—melting enthalpy.

**Table 2:** Density of extruded and hot pressed straw/LLDPE composite.

Manufactured Process	Extrusion	Hot Pressing
Density/(g·cm^−3^)	1.31	0.83
